# Management of Peripheral Cemento-Ossifying Fibroma

**DOI:** 10.7759/cureus.107207

**Published:** 2026-04-17

**Authors:** Mythili Sabesan, Sharada T Rajan, Vijaya Nirmala, Deepak M Ravindran, Brittilla Sharon

**Affiliations:** 1 Oral Pathology and Microbiology, Sri Ramachandra Institute of Higher Education and Research, Chennai, IND; 2 Oral Pathology, Sri Ramachandra Institute of Higher Education and Research, Chennai, IND; 3 Periodontology, Sri Ramachandra Institute of Higher Education and Research, Chennai, IND

**Keywords:** minor oral surgery, oral biopsy, oral health, oral & maxillofacial pathology, perio pathogens, perio plastic surgeries

## Abstract

Peripheral cemento-ossifying fibroma (PCOF) is a rare, reactive osteogenic neoplasm that presents as an epulis-like growth with unknown pathogenesis. This lesion commonly affects the younger generation. Frequently associated with the maxilla, it is more common in the anterior region. Here, we present a rare case of a 54-year-old male patient with a painless gingival swelling in the mandible for a period of one year. Prompt diagnosis through complete radiographic and histopathologic examination is critical for proper diagnosis of this lesion. Proper surgical removal with complete curettage is extremely important for the prevention of recurrence.

## Introduction

Peripheral cemento-ossifying fibroma (PCOF) is a slow-growing, reactive lesion occurring on the gingiva containing mineralized product arising from the periodontal ligament or the periosteal cells [1,2]. The 1992 World Health Organization groups under a single entity, cemento-ossifying fibroma, two histologic patterns-mineralization resembling bone (ossifying fibroma) and cementum (cementifying fibroma) that may be clinically and radiographically indistinguishable. PCOF is a rare lesion that is well demarcated and occasionally encapsulated, showing fibrous maturation and calcifications [3]. The common etiologic factors include trauma and irritants from the bacterial plaque, calculus, orthodontic appliance, overhanging restorations, and improper fit crowns [4]. PCOF reports roughly for 3.1% of all tumors and 9.6% of gingival lesions. Although both genders are affected, reports state a higher incidence of this lesion in women [5]. PCOF demonstrates as a sessile or pedunculated mass arising from the interdental papilla [6]. It is a slow-growing mass usually of smaller size < 2 cm in dimension but rarely presents as an enlarging mass on the gingiva progressively leading to facial deformity [7]. Tooth migration may be noted in some cases. Radiographic presentation does not indicate any bony involvement; however, in some cases, superficial erosion might be noted [8,9]. These lesions are treated by surgical excision, and diagnosis is confirmed by histopathological examination. Here, we present a report of a rare case of PCOF in the mandibular gingiva of the posterior tooth.

## Case presentation

**Figure 1:**
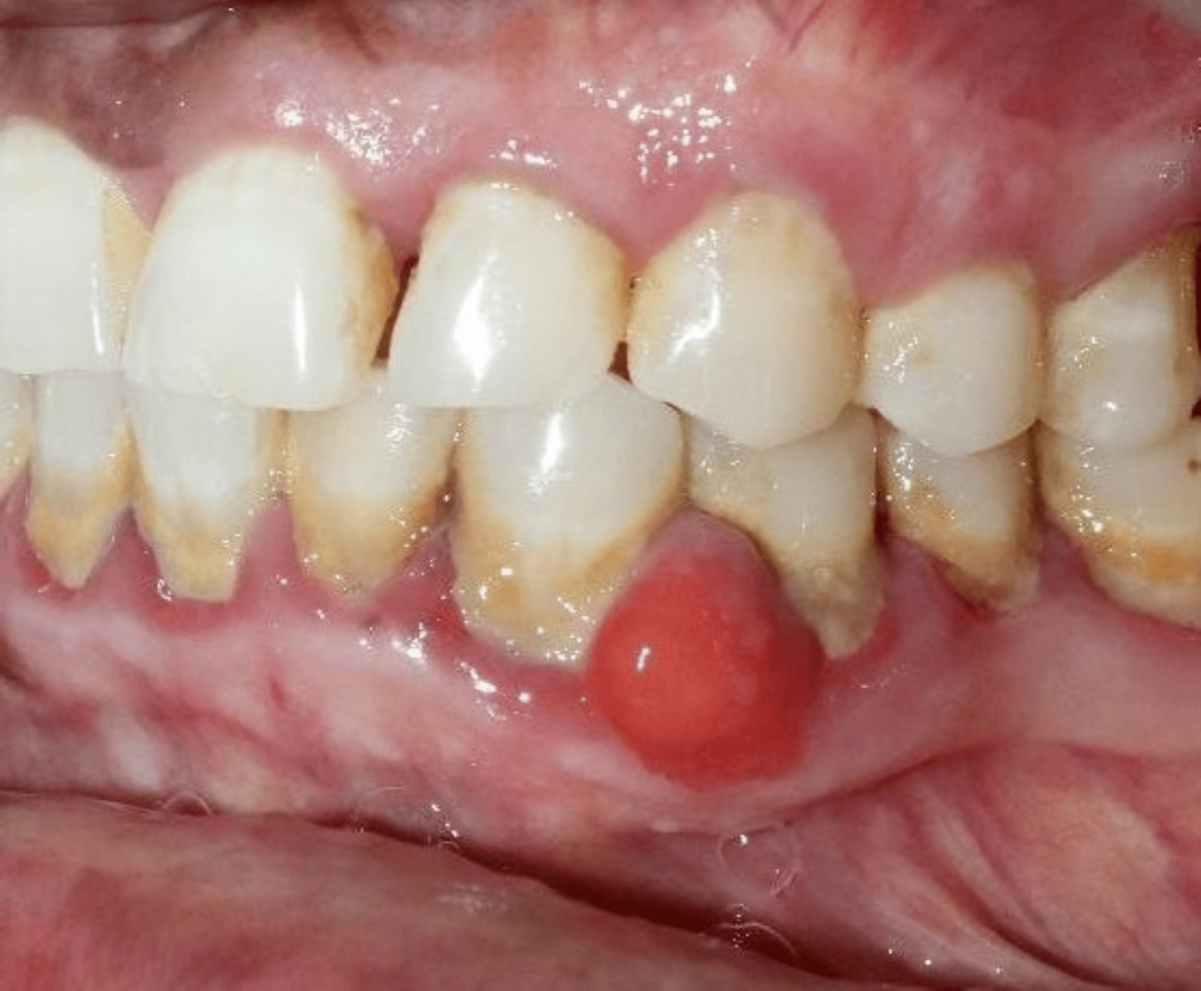
Intraoral swelling evident in the interdental papilla of the 33-34 region

A 54-year-old male patient reported to the outpatient Department of Periodontics, Sri Ramachandra Dental College and Hospital, Sri Ramachandra Institute of Higher Education and Research (SRIHER), Chennai, India, with a chief complaint of a painless swelling in the lower right front tooth region (Figure 1). The swelling had been slowly increasing in size for the past one year. Additionally, the patient complained of bleeding and slight mobility of the teeth. The patient’s past dental, medical, and habit histories were unremarkable.

Intraoral examination revealed a well-circumscribed, erythematous sessile growth on the interdental region of 33-34, measuring about 1 cm × 1.5 cm (Figure 1), which was firm and non-tender on palpation. The swelling was non-fluctuant and did not blanch under pressure. The nodes were non-palpable and non-tender. The presence of plaque and calculus was evident.

**Figure 2:**
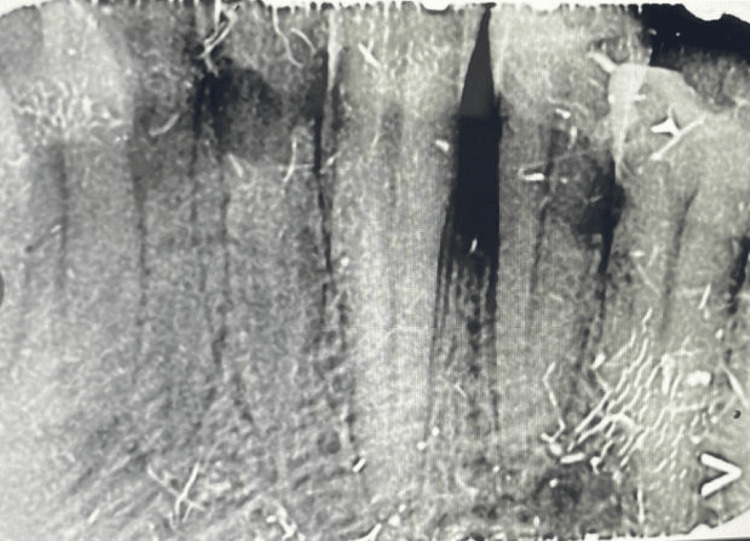
Intraoral periapical radiograph showing interdental bone loss in the 32-33 region

Radiographic examination revealed an interdental bone loss in relation to the 32 and 33 region (Figure 2).

**Figure 3:**
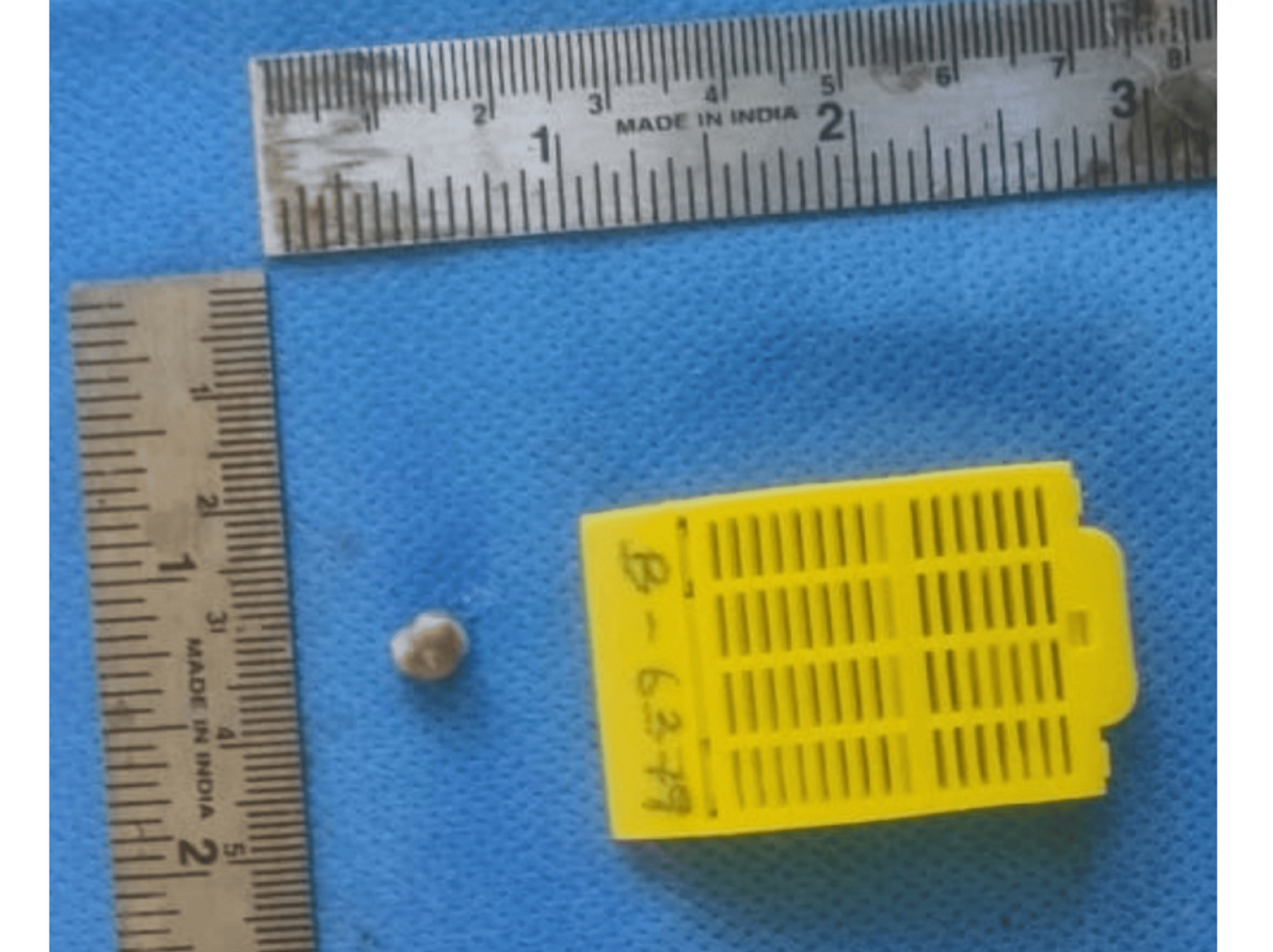
Excised tissue was soft in consistency, brown in color, and irregular in shape, measuring 0.8 × 0.6 × 0.4 cm

The patient underwent complete blood investigations and found that all readings were within normal limits. Based on the clinical and radiographic examination, a provisional diagnosis of pyogenic granuloma was made. The differential diagnoses considered were fibroma, PCOF, and peripheral giant cell granuloma. The patient underwent oral hygiene prophylaxis, and the swelling was excised under local anesthesia using a scalpel and a blade. Hemostasis was achieved, and the surgical area was covered with a periodontal dressing (GC Coe-Pak; Tokyo, Japan). The excised tissue was sent for histopathological diagnosis (Figure 3).

**Figure 4:**
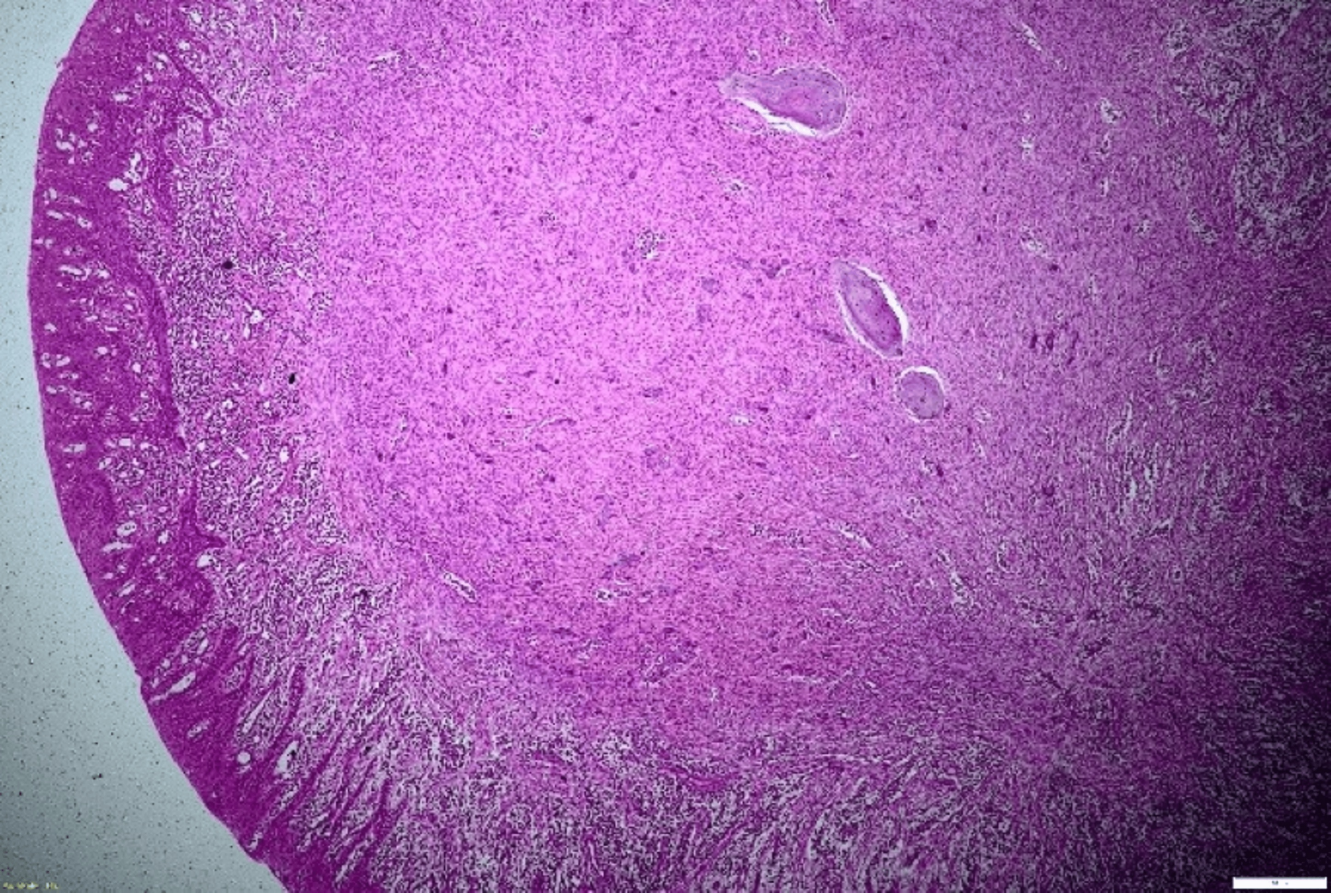
Hematoxylin and eosin (H&E) staining: a x4 view section showing a fibrocellular stroma lined by parakeratinized stratified squamous epithelium

**Figure 5:**
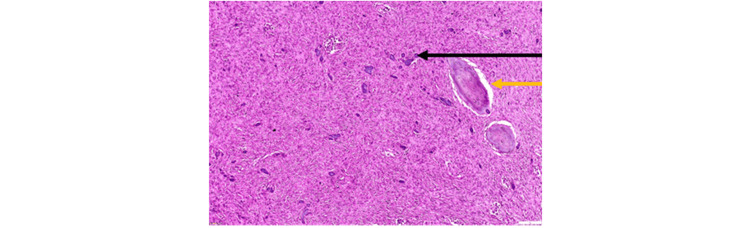
x10 view: arrows showing osteoid tissue and cementoid

**Figure 6:**
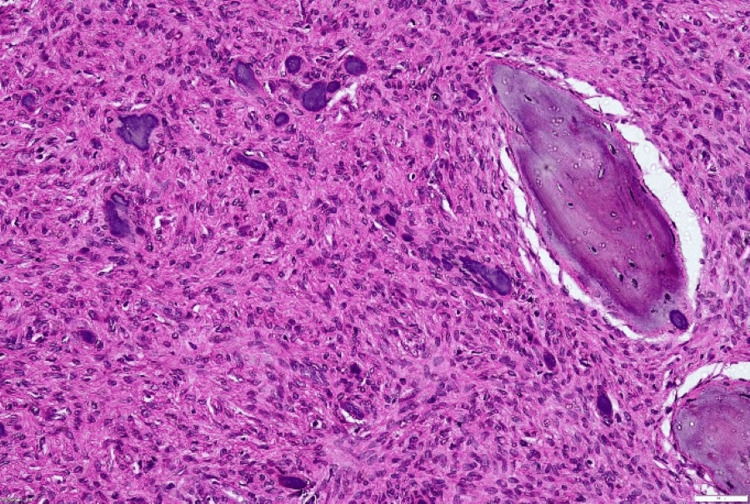
Hematoxylin and eosin (H&E) staining, x40: round to ovoid basophilic cementum-like calcifications and osteoid tissue

On histopathological examination (Figures 4, 5), the given section showed parakeratinized stratified squamous epithelium with long and slender rete ridges. The underlying connective tissue revealed a fibrocellular stroma composed of plump fibroblasts, collagen fibers, blood vessels, and inflammatory cell infiltrate. Admixed in the connective tissue were a few round to ovoid cementum-like calcified structures. Also, areas of osteoid calcifications containing osteocytes and lined by osteoblasts were seen. These features were suggestive of PCOF (Figure 6).

**Figure 7:**
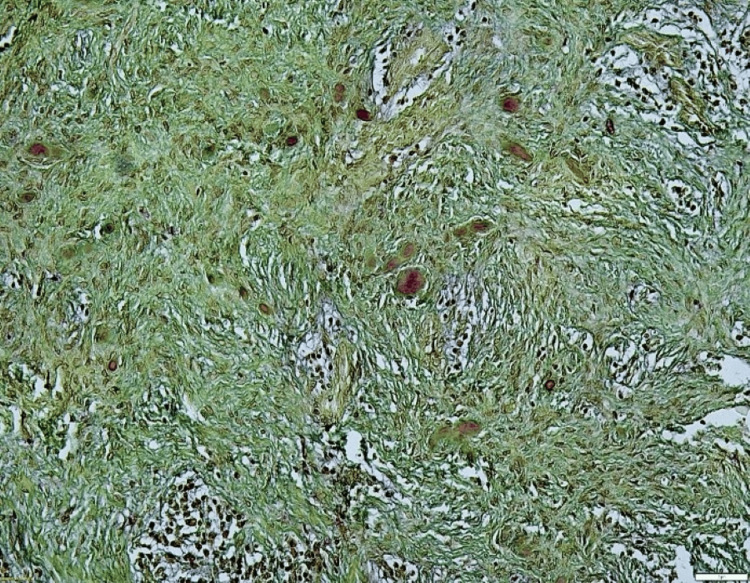
Modified Gallego’s staining, x4 view: section showing a fibro stroma containing cementoid tissue

**Figure 8:**
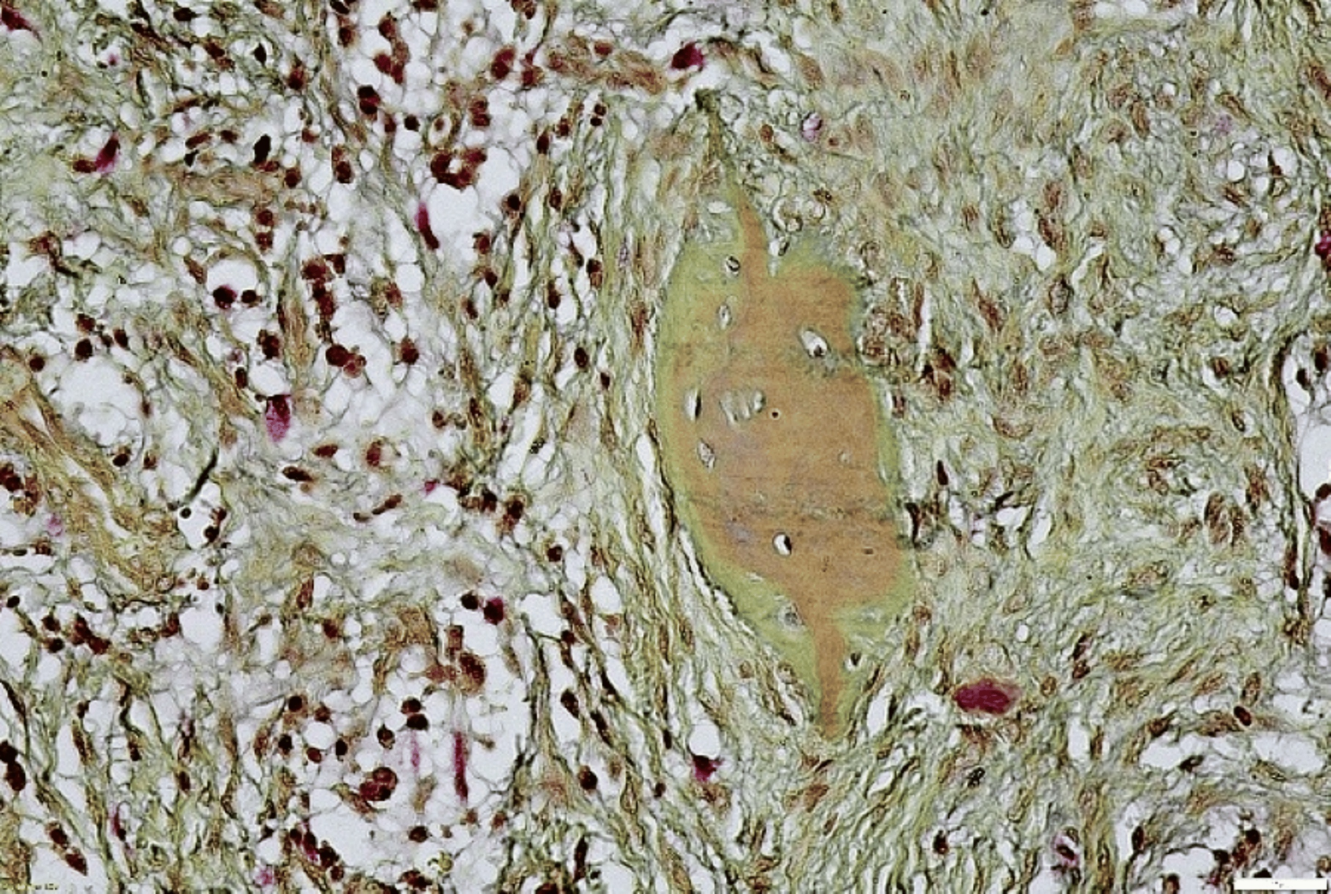
Modified Gallego’s staining, x40: round to ovoid basophilic cementum-like calcifications and osteoid tissue

Further, we went on to determine the nature of the calcified structures of the lesion. Modified Gallego’s stain was performed to aid in the differential staining of the hard tissues. Microscopic features revealed that osteoid material stained green while the cementoid substance stained red (Figures 7, 8). The patient has been under a 1.5-year follow-up with no evidence of recurrence.

## Discussion

PCOFs were initially placed under the category of mesenchymal odontogenic tumors in the WHO classification (2017) but are traditionally explained under benign fibro-osseous lesions with a global incidence of less than 3.1% [1,2]. They are more frequently seen in the interdental papillae of the anterior maxillary region, with a slight female disposition attributed to the effect of hormones and Caucasoid race predilection of young adults. This is in stark contrast to our current case in discussion [2,3,10]. The pathogenesis of PCOF has ranged from reactive to neoplastic growth. While usually they have been associated with the presence of local irritants such as plaque and calculus, this was also evident in our case. With an unclear etiology, PCOFs are thought to arise from the periodontal ligament cells owing to their preponderance in the gingival areas [11]. Pereira et al. have shown that deregulation of a signaling gene in the wnt/β-catenin pathway plays a role in the pathogenesis of COF [12].

A global perplexity exists in association with the naming of the lesion as “ossifying” when bone tissue predominates and “cementifying” when spheroidal basophilic calcifications resembling cementum are seen in histopathology. When both types are seen, it is then referred to as the “cemento-ossifying” type. In the current case, the authors attempted to analyze the differentiation of both types of calcifications that were present in the lesion. Modified Gallego’s staining was performed and observed under the microscope. While the bony osteoid calcifications stained green, the basophilic cementoid depositions stained a reddish hue, thereby confirming the differentiation of the type of calcification. Similar results were observed in a study by Tamgadge et al. [13].

Differentiation of calcified tissues is essential to obtain a clearer histopathological picture and pathogenesis of the disease process. In the current case of PCOF, the identification of both cementoid and osteoid calcifications was confirmed by using a differential special stain, thereby supporting the theory of mesenchymal stem cells from the periodontal ligament that can produce a varied mixture of calcifications in the lesional tissue. In some cases, pathologists can occasionally encounter difficulty in differentiating the calcified tissues based on morphological features using the simple routine hematoxylin and eosin stain, which can be circumvented easily using the differential staining of modified Gallego (Table 1).

**Table 1:** Differential staining by modified Gallego’s stain H&E: hematoxylin and eosin

Types of stains	Bone	Cementum
H&E stain	Basophilic	Basophilic
Modified Gallego’s stain	Yellowish green	Pinkish red

While removal of local irritants by scaling is performed, surgical excision, including the periodontal ligament that is involved, along with the periosteum, is the treatment of choice. Given its recurrent nature, frequent follow-up is essential [4,10,11].

## Conclusions

This case report depicts a rare benign odontogenic mesenchymal tumor that outlines the clinical, histopathological, and treatment aspects of the lesion, along with an aid to the histological differentiation of the calcified structures in the lesional tissue that can help in the proper diagnosis of the disease, thereby supporting treatment aspects. Modified Gallego’s stain can be used as a potential practical tool in diagnostic aspects of differentiating the hard tissue structures that can throw an insight into the pathogenesis and theranostic aspects of the lesion. A thoroughly pre-planned surgery with removal of local etiological factors can help in both eliminating such recurrences as well as improving oral hygiene and esthetics.
